# Identification of Multiple Proteins Coupling Transcriptional Gene Silencing to Genome Stability in *Arabidopsis thaliana*

**DOI:** 10.1371/journal.pgen.1006092

**Published:** 2016-06-02

**Authors:** Christopher J. Hale, Magdalena E. Potok, Jennifer Lopez, Truman Do, Ao Liu, Javier Gallego-Bartolome, Scott D. Michaels, Steven E. Jacobsen

**Affiliations:** 1 Department of Molecular, Cell and Developmental Biology, University of California at Los Angeles, Los Angeles, California, United States of America; 2 Center for Precision Diagnostics, University of Washington, Seattle, Washington, United States of America; 3 Department of Biology, Indiana University, Bloomington, Indiana, United States of America; 4 Howard Hughes Medical Institute, University of California at Los Angeles, Los Angeles, California, United States of America; Gregor Mendel Institute of Molecular Plant Biology, AUSTRIA

## Abstract

Eukaryotic genomes are regulated by epigenetic marks that act to modulate transcriptional control as well as to regulate DNA replication and repair. In *Arabidopsis thaliana*, mutation of the ATXR5 and ATXR6 histone methyltransferases causes reduction in histone H3 lysine 27 monomethylation, transcriptional upregulation of transposons, and a genome instability defect in which there is an accumulation of excess DNA corresponding to pericentromeric heterochromatin. We designed a forward genetic screen to identify suppressors of the *atxr5/6* phenotype that uncovered loss-of-function mutations in two components of the TREX-2 complex (AtTHP1, AtSAC3B), a SUMO-interacting E3 ubiquitin ligase (AtSTUbL2) and a methyl-binding domain protein (AtMBD9). Additionally, using a reverse genetic approach, we show that a mutation in a plant homolog of the tumor suppressor gene BRCA1 enhances the *atxr5/6* phenotype. Through characterization of these mutations, our results suggest models for the production *atxr5 atxr6*-induced extra DNA involving conflicts between the replicative and transcriptional processes in the cell, and suggest that the *atxr5 atxr6* transcriptional defects may be the cause of the genome instability defects in the mutants. These findings highlight the critical intersection of transcriptional silencing and DNA replication in the maintenance of genome stability of heterochromatin.

## Author Summary

In eukaryotic genomes cellular processes such as transcription and replication need to be tightly controlled in order to promote genomic stability and prevent deleterious mutations. In *Arabidopsis thaliana*, two redundant histone methyltransferases, ATXR5 and ATXR6, are responsible for the deposition of a silencing epigenetic mark, histone H3 lysine 27 monomethylation. Loss of ATXR5/6 results in transcriptional activation of transposable elements (TEs), upregulation of DNA damage response genes and a genomic instability defect characterized as an excess of DNA corresponding to heterochromatin regions. Using a genetic screen, we sought to find suppressors of the *atxr5/6* phenotype, and interestingly, we identified multiple genes implicated in general transcriptional activity. Through genomic characterization of the mutants our data suggest a model where transcriptional silencing of heterochromatin during S-phase is required for proper replication and maintenance of genome stability. These findings emphasize the important relationship between chromatin, transcriptional control and replication in the maintenance of genome stability in a eukaryotic system and identify new players involved in these processes.

## Introduction

The genome represents a biological entity that is necessarily static yet retains a level of plasticity. Cells must faithfully replicate their genomes to avoid deleterious mutations, but also must be responsive to external stimuli. Eukaryotes have evolved multiple layers of epigenetic regulation that allow the genome to respond to environmental and developmental changes as well as provide a level of genome defense against parasitic genetic elements such as transposons. While epigenetic and replication fidelity pathways have traditionally been thought to be independent, multiple lines of evidence have recently implicated epigenetic mechanisms in the regulation of DNA replication [1–4].

In *Arabidopsis thaliana*, we previously identified two redundant histone methyltransferases, ATXR5 and ATXR6 (referred to in the aggregate as ATXR5/6), that are responsible for monomethylating lysine 27 of histone H3 (H3K27me1) [5]. Loss of these methyltransferases in the *atxr5/6* double mutant leads to a severe loss of transcriptional silencing at repetitive transposable elements (TEs) [5,6]. The *atxr5/6* mutants also display an unusual phenotype, wherein heterochromatin regions of the Arabidopsis genome exhibit an aberrant gain in DNA copy number (here referred to simply as over-replication). The over-replication phenotype appears to be mainly in cells which have undergone endoreduplication, a form of cell cycle without mitosis frequently observed in terminally differentiated cells [7]. The regions producing extra DNA are highly repetitive and carry epigenetic marks characteristic of silent chromatin such as DNA methylation and H3K27me1, and largely overlap with the pericentromeric regions transcriptionally derepressed in the *atxr5/6* mutant.

We previously showed that mutations that strongly reduce DNA methylation in an *atxr5/6* mutant background suppress the over-replication phenotype of *atxr5/6* [6], suggesting that the heterochromatic nature of these regions is necessary to engender the gain in DNA copy number phenotype of the *atxr5/6* mutant. In addition, while the DNA methylation mutants suppressed the over-replication phenotype of *atxr5/6* mutants, they actually enhanced the transcriptional derepression phenotype [6]. Thus, the extra DNA phenotype and the transcriptional silencing phenotypes were decoupled in these mutants, showing that the extra DNA production in *atxr5/6* is not required for the aberrant transcriptional activation of transposable elements.

In order to better understand the relationship between the *atxr5/*6 silencing and DNA copy number phenotypes, we carried out an extensive analysis of the *atxr5/6* transcriptome, and compared this with the transcriptome of plants undergoing DNA damage. We also identified a number of modulators of the *atxr5/6* phenotype by forward and reverse genetics approaches. Most notably, we isolated mutations in several genes, including those encoding members of the TREX-2 complex, a methyl-binding domain protein, and a SUMO dependent E3 ligase that suppressed the transcriptional defects in *atxr5/6* mutants together with the genomic instability defects. Furthermore, we found that mutation of a gene involved in DNA repair and replication fork stability, *BRCA1*, enhanced both the *atxr5/6* transcriptional and over-replication phenotypes. These results suggest a very close relationship between the loss of transcriptional silencing in *atxr5/6* mutants and over-replication, consistent with a model wherein inappropriate transcription in *atxr5/6* conflicts with the normal replication of heterochromatin to cause genomic instability.

## Results

### Loss of transposon silencing in *atxr5/6* mutants is tissue specific and correlates with extra DNA at pericentromeric heterochromatin

**Fig 1:**
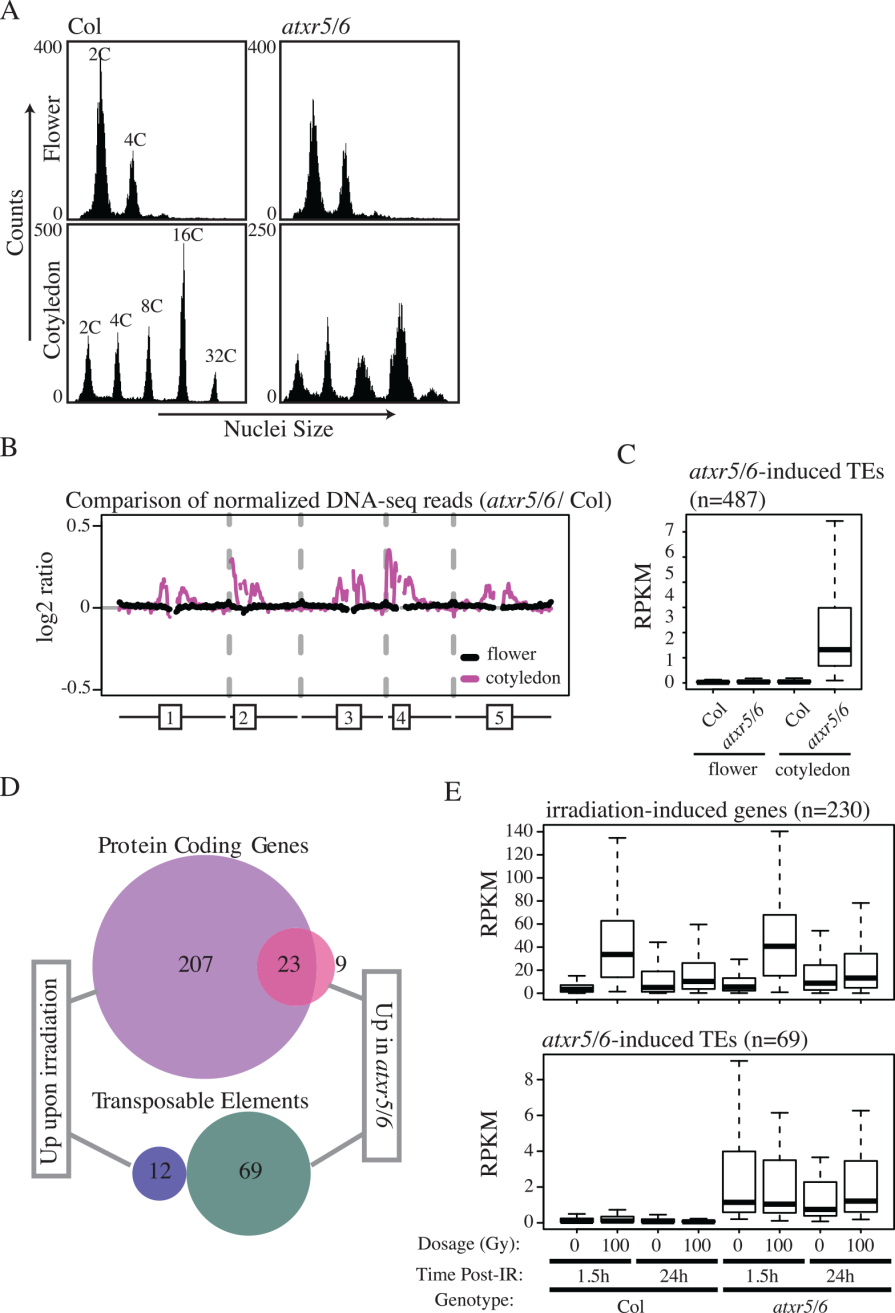
Transcriptional silencing defects in an *atxr5/6* mutant are strongly correlated with over-replication defects and are not induced by DNA damage. (A) Flow cytometry profiles of wild type plants (Col) and *atxr5/6* mutants for flower and cotyledon tissue with ploidy levels labeled for Col profiles. Excess DNA production is seen as a broadening of the peaks in *atxr5/6* cotyledons compared to wild type. (B) Chromosomal views of DNA sequencing read ratio of *atxr5/6* mutants compared to Col for flower and cotyledon tissues with diagrammatic representations of the Arabidopsis chromosomes shown below with boxes identifying pericentromeric heterochromatin. Gaps in the plot represent areas of low coverage. (C) Boxplot of RNA-seq RPKM values for *atxr5/6*-induced TEs from cotyledon tissue in floral and cotyledon tissue. (D) Venn diagrams describing the relationship between genes *de novo* identified as up-regulated in the irradiation and *atxr5/6* mutant transcriptomes. (E) Boxplots showing the behavior of *atxr5/6*- irradiation-induced protein coding genes (top) and TEs (bottom) for various radiation dosages, time points, and genotypes.

We previously observed that the *atxr5/6* mutants produce excess DNA corresponding to heterochromatin regions, also referred to as over-replication, that was most obvious in nuclei that had undergone endoreduplication [7], a process that is systemic in many tissue types of Arabidopsis and roughly correlates with tissue age [8]. We sought to test whether the transcriptional silencing defect of *atxr5/6* mutants is similarly confined to specific tissues. To do this we analyzed two tissue types with different levels of endoreduplication, immature floral tissue that shows very low levels of endoreduplication, and cotyledons (embryonic leaves) that are very highly endoreduplicated. These tissues were analyzed by flow cytometry (Fig 1A) and whole-genome re-sequencing to confirm the state of over-replication in *atxr5/6* (Fig 1B). Consistent with previous profiling of nuclei of different ploidy levels [7], we found an increase in DNA copy number in *atxr5/6* mutants in cotyledons (Fig 1A) that was localized to regions of pericentromeric heterochromatin (Fig 1B). The excess DNA was absent in floral tissue of *atxr5/6* mutants by both flow cytometry and sequencing analysis (Fig 1A and 1B).

We next performed RNA sequencing (RNA-seq) of flower and cotyledon tissue. We observed a reduction of the *atxr5/6* transcriptional silencing defect in flowers (Fig 1C) that paralleled the lack of the extra DNA phenotype. While we were able to identify 487 TEs up-regulated in *atxr5/6* cotyledon tissue relative to wild type (S1 Table), we found only 5 TEs up-regulated in floral tissue (S1 Table) and the TEs identified in cotyledon tissue showed greatly reduced transcription in *atxr5*/6 flowers (Fig 1C). Together these results suggest that the co-occurrence of the transcriptional defect and the over-replication defect in the *atxr5/6* mutant may be connected and specific to endoreduplicated tissues.

### The *atxr5/6* transcriptome resembles a genome undergoing constitutive, low-level DNA damage

We previously observed that, consistent with the over-replication phenotype in the *atxr5/6* mutants that is likely to cause genomic instability, several genes involved in the homologous recombination (HR) DNA repair pathway were up-regulated in *atxr5/6* [6]. To assess the extent of DNA damage pathway activation in the mutants, we generated RNA-seq data from wild type seedlings that had undergone gamma-irradiation, which is known to generate robust activation of DNA repair pathways. From this analysis, we identified 230 protein-coding genes that were activated 90 minutes post-irradiation (S2 Table). This identified gene set significantly overlapped (S1 Fig) with a previously published set of gamma-irradiation responsive genes identified by microarray analysis [9], though the RNA-seq method identified a larger set of genes than the microarray-based approach. Using this gene set we were able to conclude that the majority of the protein-coding genes up-regulated in *atxr5/6* seedlings belong to genes that are upregulated upon irradiation (Fig 1D), implying that the *atxr5/6* protein-coding gene expression changes mainly reflect a response to DNA damage. Furthermore, upon irradiation of *atxr5/6* mutants, we observed a robust upregulation of the same DNA damage genes that were up-regulated upon irradiation in wild type plants (Fig 1E), indicating that DNA damage response signaling is intact in *atxr5/6* mutants. Therefore, we concluded that the excessive DNA phenotype in *atxr5/6* mutants is not due to a generalized failure to induce DNA damage pathways.

Using the seedling RNA-seq datasets, we also defined TEs up-regulated upon irradiation (Col 100Gy compared to Col 0Gy) as well as due to the *atxr5/6* mutations (*atxr5/6* 0Gy compared to Col 0Gy) (Fig 1E). We identified fewer TEs up in the *atxr5/6* seedling data (n = 69) than the cotyledon datasets (n = 487), likely due to the heterogeneous nature of tissues from whole seedlings that have fewer endoreduplicated nuclei than do cotyledons. Importantly, we failed to observe a large increase in transposon expression post-irradiation (Fig 1E). This was true at the 90 minute time point as well as at 24 hours post-irradiation (Fig 1E). These results indicate that the transposon silencing defect we observe in *atxr5/6* mutants is most likely not simply a consequence of the DNA damage induced in those mutants.

### Mutation of the Arabidopsis BRCA1 homolog (AtBRCA1) enhances the silencing and over-replication defects of *atxr5/6* mutants

**Fig 2:**
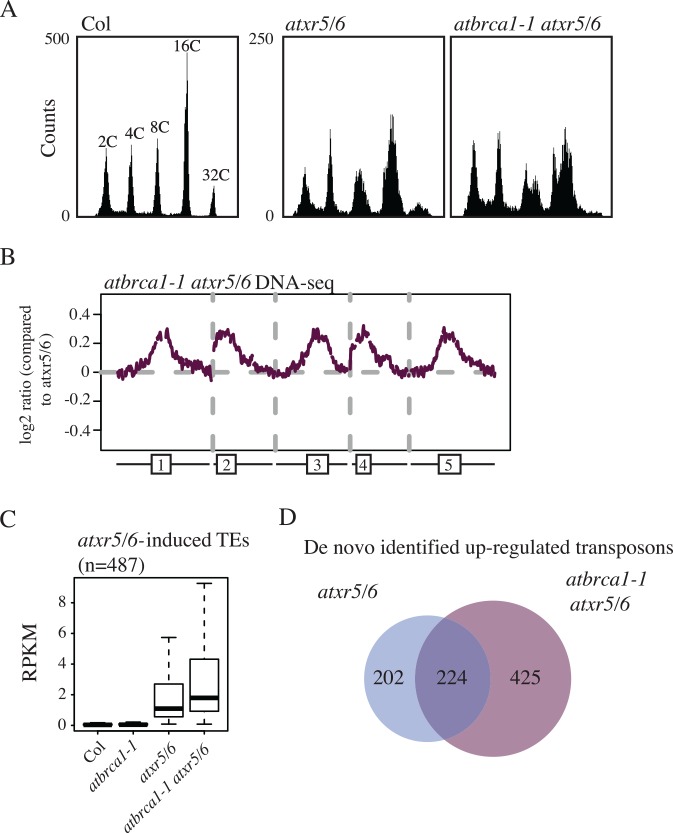
Loss of the Arabidopsis *BRCA1* homolog enhances the *atxr5/6* extra DNA and transcriptional silencing phenotypes. (A) Flow cytometry of cotyledon tissue for *Col*, *atxr5/6*, and *atbrca1-1 atxr5/6* lines. Col and *atxr5/6* data is same as shown in Fig 1A and is shown here for comparison. (B) Chromosomal views as in Fig 1B comparing *atbrca1-1 atxr5/6* sorted 16C DNA-seq to sorted 16C reads from *atxr5/6*. (C) Boxplot of RNA-seq RPKM values for *atxr5/6*-induced TEs in cotyledon tissue for genotypes derived from the listed genotype. (D) Venn diagram of TEs *de novo* identified from *atxr5/6* and *atbrca1-1 atxr5/6*.

Given our observation that *atxr5/6* mutants show a generalized activation of DNA damage response pathways (especially HR genes) we sought to assess the effect of loss of DNA damage response gene in *atxr5/6* mutants. To do this we generated *atbrca1 atxr5/6* triple mutants. In mammals BRCA1 is a well characterized HR pathway protein important in maintaining genome stability with additional functions in cell-cycle check point and transcriptional regulation [10–13]. In plants *AtBRCA1* has been shown to be necessary for efficient DNA repair [14] and is among the most highly up-regulated genes in an *atxr5/6* mutant [6]. Interestingly, we found that the *atbrca1 atxr5/6* mutants exhibited an enhancement of *atxr5/6*-induced extra DNA phenotype by flow cytometry and whole-genome sequencing of sorted 16C nuclei (Fig 2A and 2B).

We used RNA-seq of cotyledons to compare wild type, *atbrca1*, *atxr5/6*, *and atbrca1 atxr5/*6 plants. The *atbrca1 atxr5/6* mutants showed a marked increase in expression of TEs identified as being overexpressed in *atxr5/6* (Fig 2C). In addition, *de novo* identification of up-regulated TEs identified a greater number of reactivated TEs in *atbrca1 atxr5/6* plants than *atxr5/6* mutants (Fig 2D). Together these results suggest that wild type BRCA1 acts to restrict the *atxr5/6* phenotype.

### Establishment of a forward genetic screen to identify factors that influence the genomic instability phenotype of *atxr5/6* mutants

In order to learn more about the biological mechanisms underlying the apparent link between the over-replication and transcription phenotypes of *atxr5/6*, we established a forward genetic screen to identify suppressors of the *atxr5/6* phenotype. We fused a GFP reporter to the promoter of the *RAD51* DNA damage response gene that is highly over-expressed in *atxr5/6* mutant. Transgenic lines carrying the RAD51 promoter-GFP fusion construct showed strong GFP fluorescence in cotyledons when in the presence of the *atxr5/6* mutations but not when crossed to wild type plants (S2A Fig). Thus, GFP fluorescence correlated with endoreduplicated tissues showing upregulated expression of transposons and extra DNA in *atxr5/6* mutants, and therefore appeared to be a suitable visual readout of the *atxr5/6* phenotype. We carried out an EMS mutagenesis of *atxr5/6 RAD51pro*::*GFP* seed (referred to as *RAD51pro*::*GFP* from this point forward) and searched for suppressors of the *atxr5/6* phenotype by screening for families segregating plants that had lost the cotyledon GFP expression (GFP-, S2B and S2C Fig).

### Identification of mutations in two components of the TREX-2 complex as suppressors of the *atxr5/6* phenotype

**Fig 3:**
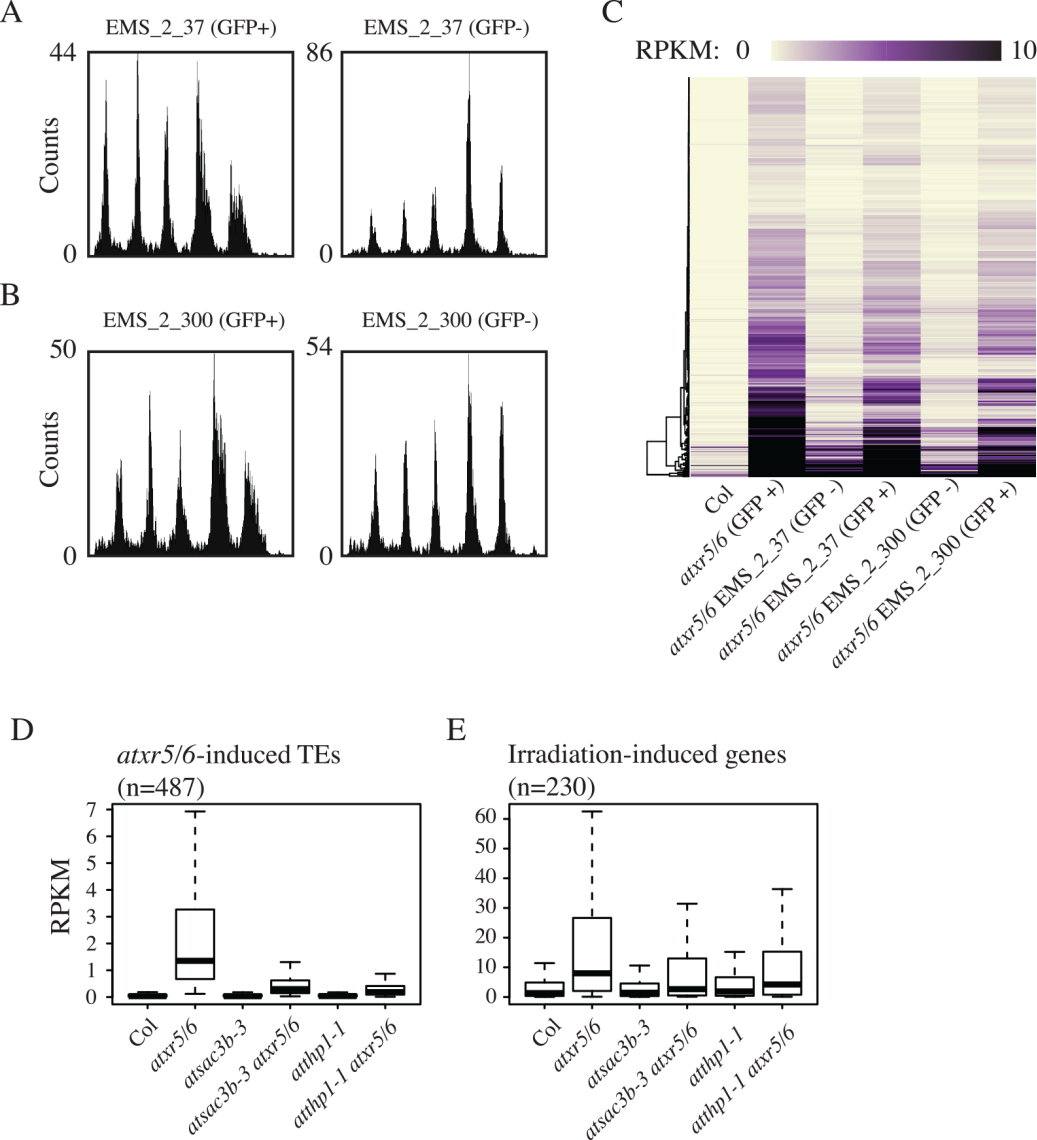
Mutations in Arabidopsis TREX-2 complex proteins suppress the transcriptional silencing and extra-DNA phenotypes of *atxr5/6* mutants. (A) Flow cytometry of M2 *atxr5/6* plants containing *RAD51pro*::*GFP* from the EMS_2_37 and (B) EMS_2_300 lines for both GFP+ and GFP- plants. (C) Heatmap of RNA-seq RPKM values over cotyledon *atxr5/6*-induced TEs for F2 EMS_2_37 or EMS_2_300 GFP+/- as well as control Col and *atxr5/6* GFP+ plants. All lines except for Col contain *RAD51pro*::*GFP* and are in *atxr5/6* background. (D) Boxplot of RNA-seq RPKM values from cotyledon tissue for identified *atxr5/6*-induced TEs (S1 Table) and (E) irradiation-induced genes (S2 Table) in TREX-2 insertional mutants.

Utilizing two M2 lines, EMS_2_37 and EMS_2_300, segregating for mutations causing loss of GFP signal (*ems_2_37* and *ems_2_300*, S2C Fig), we performed flow cytometry on the GFP- plants as well as GFP+ plants from the same M2 family. For both the EMS_2_37 and EMS_2_300 lines, we observed clear suppression of the *atxr5/6* extra DNA defect for GFP- plants (Fig 3A and 3B). We backcrossed the GFP- *ems_2_37* and *ems_2_*300 mutants to the *atxr5/*6 line to confirm the function of the GFP protein in the F1 generation and to confirm the resegregation of GFP- plants in the resultant F2 generation (S2B Fig). We performed RNA-seq on cotyledons from the GFP- plants from this F2 generation as well as the GFP+ segregants from the same families. We found a striking reduction of TE expression in the GFP- plants as compared to both the GFP+ siblings and the starting *RAD51pro*::*GFP* line (Fig 3C), revealing that the suppression of the extra DNA in *atxr5/6* by these mutants was accompanied by suppression of the *atxr5/6* transcriptional silencing defect. This result is markedly different from the previously characterized suppression of *atxr5/6*-induced over-replication by DNA methylation mutants [6], where the loss of DNA methylation caused an increase in TE expression while suppressing the generation of extra DNA in *atxr5/6* mutants.

We utilized EMS-induced mutations identified in RNA-seq datasets to map the *ems_2_37* and *ems_2_300* mutations (S3A and S3B Fig; S1 Text). The *ems_2_37* mutation mapped to a splice site mutation at *AtSAC3B* (*At3g06290*, S3C–S3F Fig), a homolog of the yeast Sac3 protein [15]. The effect of the *ems_2_37* mutation on *AtSAC3B* transcript splicing could be verified in the RNA-seq data, which showed clear intron retention relative to the control lines (S3C Fig). The *ems_2_300* mutation was found to map to a nonsense mutation in *AtTHP1* (*At2g19560*, S3F Fig), the homolog of yeast Thp1. AtSAC3B and AtTHP1 have been found to interact, analogously to their yeast homologs, in a complex termed TREX-2 [15]. The TREX-2 complex has been characterized in multiple systems as facilitating gene expression and RNA export from the nucleus via nuclear pore complexes (NPCs) [16–19]. Furthermore, the TREX-2 complex has also been found to act with transcribing RNA polymerase complexes to prevent the formation of deleterious transcriptional intermediates such as R-loops [20–22].

The molecular identities of *ems_2_37* and *ems_2_300* were confirmed by whole genome resequencing of the EMS lines to confirm the mapping of the EMS alleles (S3D and S3E Fig), as well as introgression of T-DNA-based insertional mutants for both *AtSAC3B* (*atsac3b-3*) and *AtTHP1* (*atthp1-1*) into an *atxr5/6* background to verify suppression of *atxr5/6* extra DNA (S4A and S4B Fig) as well as *atxr5/*6-induced TE gene expression (Fig 3D). We also observed a reduction in expression of irradiation-induced genes in the *trex*-2 *atxr5/*6 triple mutants relative to *atxr5/6*, consistent with the suppression of the extra DNA and its relationship to the DNA damage response (Fig 3E). The RNA-seq analysis of *atsac3b-3* and *atthp1-1* single mutant cotyledon tissue revealed no gain in transcription for *atxr5/6*-induced TEs (Fig 3D) and *de novo* calling of up-regulated TEs in the single mutants identified only 2 and 4 TEs respectively. Finally, we verified the identities of the genes by performing complementation tests between the insertional mutants and EMS alleles (S4C Fig). We therefore renamed *ems_2_37* and *ems_2_300* as *atsac3b-4* and *atthp1-5* respectively. Subsequently we identified another EMS line, EMS_2_209, carrying a nonsense mutation in AtSAC3B (S3F Fig) by whole-genome re-sequencing and confirmed the identity of this mutation (renamed *atsac3b-5*) via non-complementation with the *atsac3b-4* EMS line (S4C Fig).

### Mutation of MBD9 suppresses the *atxr5/6* phenotype

**Fig 4:**
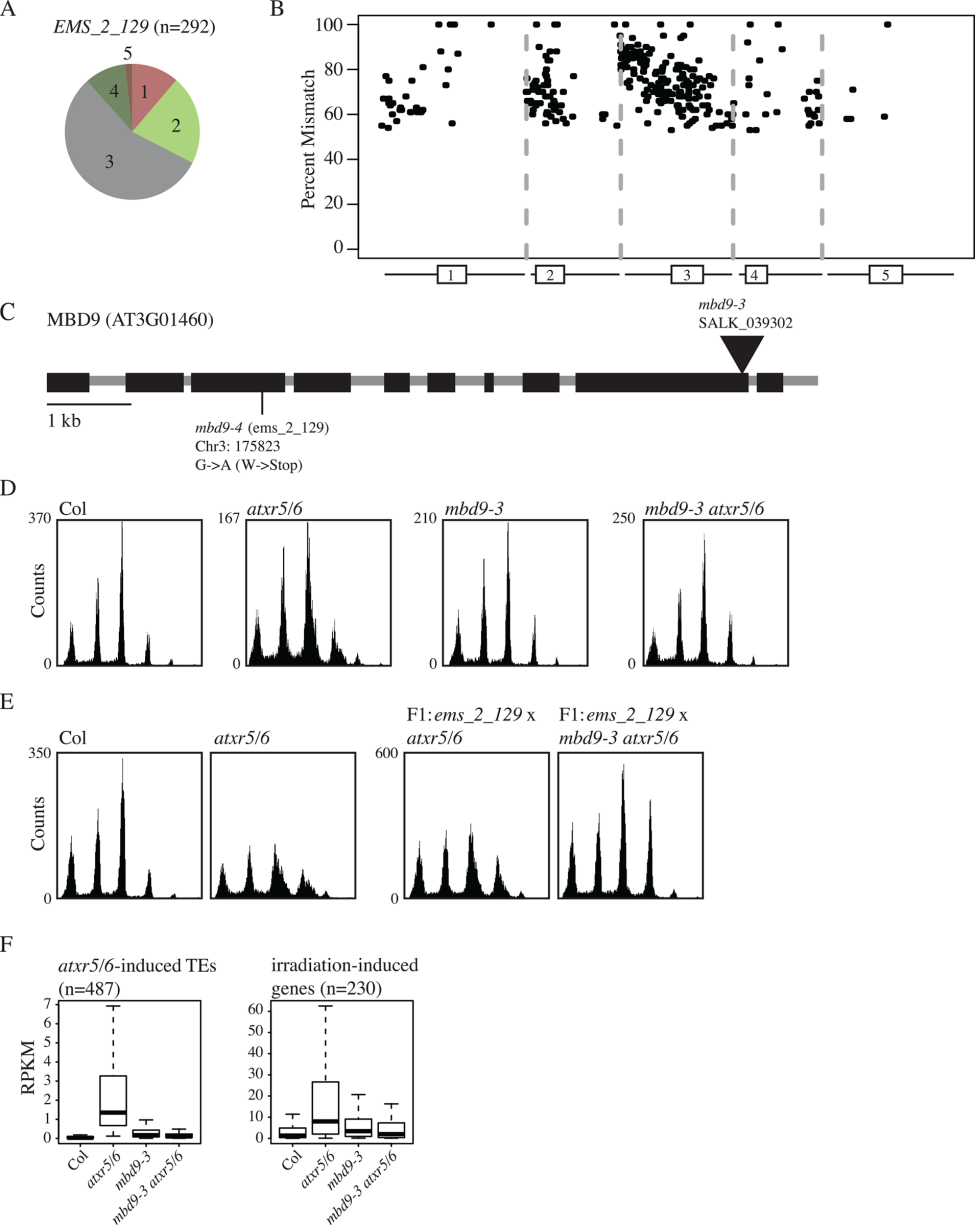
The *ems_2_129* mutation which suppresses the transcriptional silencing and extra-DNA phenotypes of *atxr5/6* mutants maps to the *MBD9* gene. (A) Pie chart and (B) chromosomal view as shown in S3A and S3B Fig showing the distribution of significantly enriched mutations in EMS_2_129 (GFP-) plants identified in DNA-seq data. (C) Gene structure of MBD9 showing the newly identified point mutation from EMS mutagenesis as well the insertional mutant (triangles) used for complementation and downstream analysis. Black boxes represent exons. (D) Flow cytometry showing that the *mbd9-3* insertional allele suppresses the *atxr5/6* extra-DNA phenotype and (E) fails to complement the *ems_2_129* EMS allele. (F) Boxplots showing the *mbd9-3* allele suppresses the *atxr5/6*-induced expression of TEs and irradiation-responsive genes.

We isolated another suppressor of the extra DNA in *atxr5/6* mutants, *ems_2_129*, which we mapped via whole-genome re-sequencing to a nonsense mutation at *mbd9* (At3g01460; Fig 4A–4D). MBD9 is a protein with a methyl CpG binding domain previously identified as a regulator of flowering time with potential roles in histone H4 acetylation [23,24]. We confirmed the identity of *ems_2_129* via introgression of the *mbd9-3* insertional allele into an *atxr5/6* background, and by performing complementation analysis (Fig 4E). RNA-seq analysis of the *mbd9-3 atxr5/6* triple mutant revealed that suppression of the *atxr5/6* extra DNA was accompanied by suppression of transcription at *atxr5/6*-induced TEs and irradiation-induced genes (Fig 4F).

### Mutation of *At-STUbL2* suppresses the *atxr5/6* phenotype

**Fig 5:**
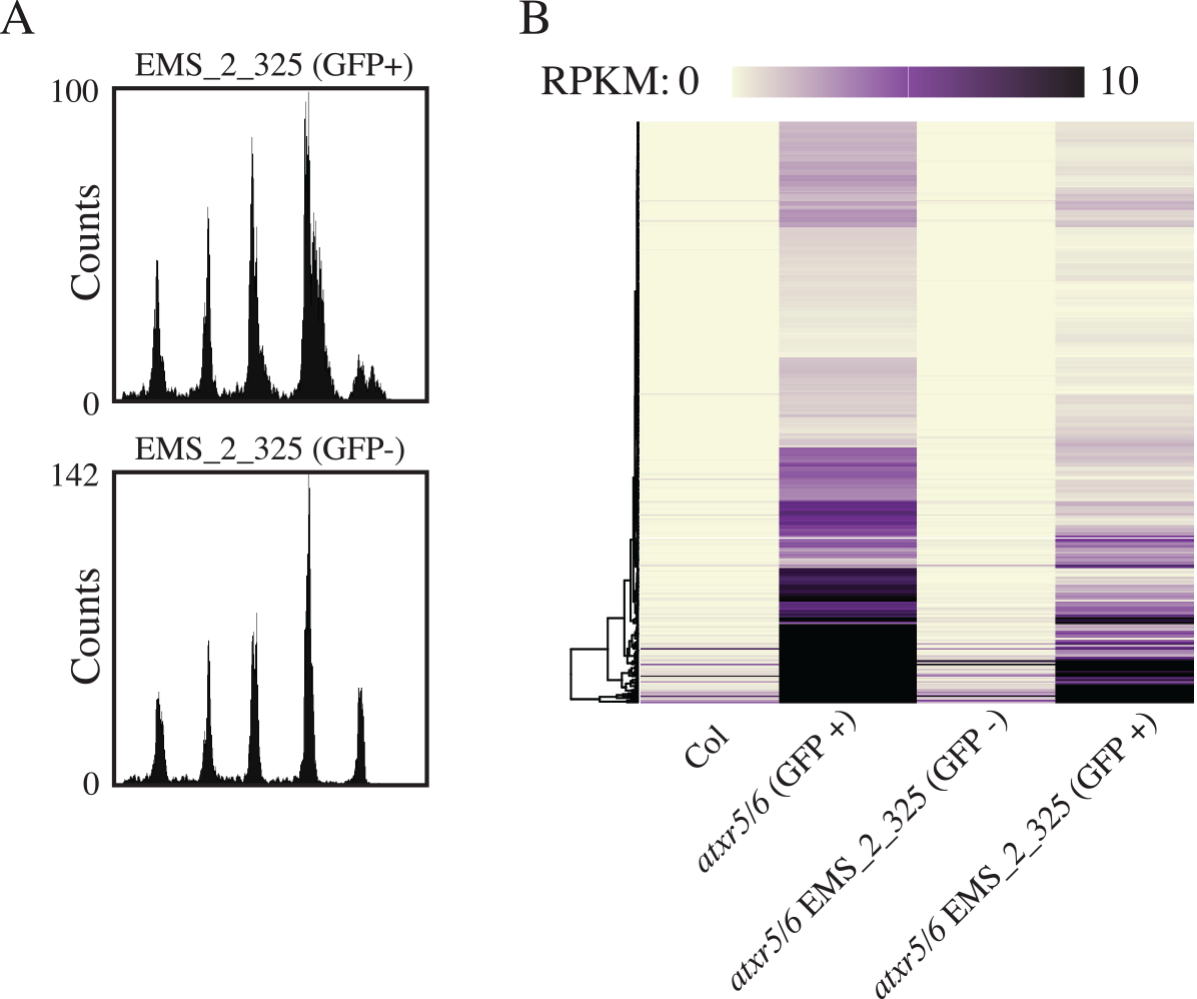
Loss of AtSTUbL2 causes suppression of the *atxr5/6* transcriptional silencing and extra-DNA phenotypes. (A) Flow cytometry of M2 *atxr5/6* plants containing *RAD51pro*::*GFP* from the EMS_2_325 line for both GFP+ and GFP- plants. (B) Heatmap for cotyledon RNA-seq, as in Fig 3C for EMS_2_325 F2 material with the same Col and *atxr5/6* GFP+ data as in Fig 3C.

A fifth mutation, *ems_2_325*, like the TREX-2 and MBD9 mutants, showed suppression of both the *atxr5/6* extra DNA and transposon over-expression phenotypes (Fig 5A and 5B). Mapping of the EMS-induced lesion by RNA-seq and subsequent whole-genome resequencing revealed a nonsense mutation in the coding region of *At1g67180* (*At-STUbL2)*, a relatively uncharacterized protein with a predicted N-terminal BRCT domain and C-terminal RING domain (S5A–S5C Fig). Complementation analysis was done using an insertional mutant (S5D–S5I Fig), the *ems_2_325* allele was renamed *stubl2-1* and the insertional *FLAG_430E03* allele was renamed *stubl2-2*. At-STUbL2 was previously identified in a yeast two-hybrid screen for proteins that bind non-covalently to SUMO, and was shown to encode a SUMO-targeted ubiquitin E3 ligase capable of complementing the growth defects of *Schizosaccharomyces pombe rfp1/rfp2* mutants [25]. In Arabidopsis, SUMO interacting proteins are highly enriched for those involved in chromatin regulation including histone and DNA methyltransferases [25]. Interestingly, At-STUbL2 was the only identified Arabidopsis STUbL protein containing the BRCT domain, which is a domain often found in proteins such as BRCA1 involved in DNA damage repair and cell cycle checkpoint control. Although the precise molecular function of *At-STUbL2* is unknown, the *At-STUbL2* RNA is co-expressed with *ATXR6* as well as DNA repair and DNA replication genes, and the MET1 and CMT3 DNA methylation proteins that are known to function during DNA replication [26–28] (S3 Table).

### Identified suppressors of *atxr5/6* show little to no alteration in DNA methylation patterns

Given our previous identification of DNA methylation mutants as suppressors of the *atxr5/6* extra DNA phenotype, we questioned whether any of the newly identified suppressors of *atxr5/6* from our forward genetic screen may have an effect on DNA methylation. To address this question, we performed whole-genome bisulfite sequencing on insertional mutants for each of the newly identified suppressors as well as wild type and *atxr5/6* controls and performed differential methylated region (DMR) discovery for the mutants as well as on a previously published *methyltransferase 1* (*met1*) dataset as a control [29]. MET1, a DNMT1 homolog, is a maintenance methyltransferase responsible for maintaining CG methylation as well as some of the non-CG cytosine methylation in the genome [29,30] and *met1* mutants suppress the *atxr5/6* extra-DNA phenotype while enhancing the TE silencing defect of *atxr5/6* mutants [6].

**Fig 6:**
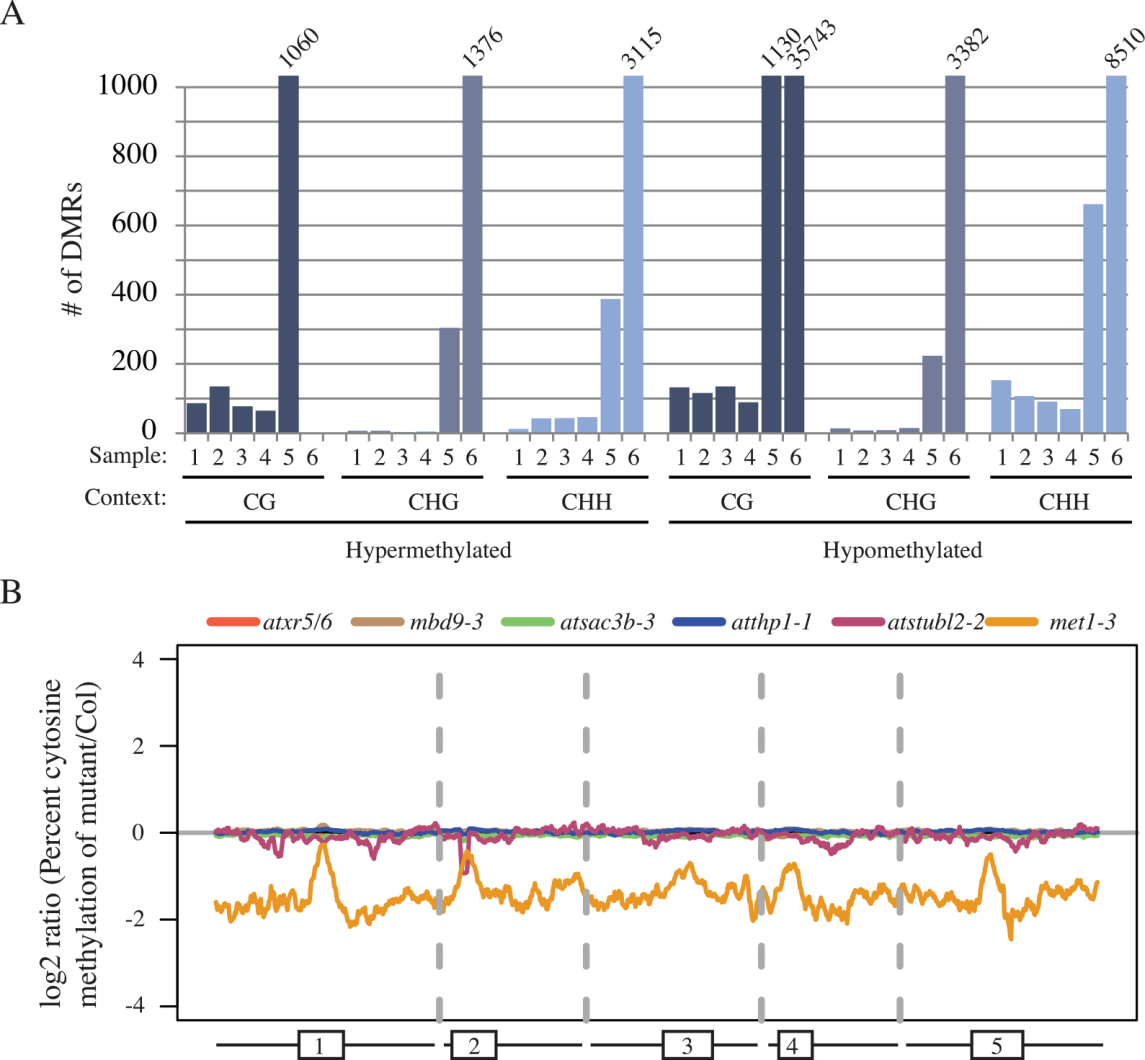
Newly identified suppressors of *atxr5/6* phenotypes do not have strong effects on DNA methylation. (A) Barplot giving the number of DMRs identified in new suppressors of *atxr5/6* with *met1* used as positive control for DMR identification (Sample 1 = *atxr5/6*, 2 = *mbd9-3*, 3 = *atsac3b-3*, 4 = *atthp1-1*, 5 = *atstubl2-2*, 6 = *met1-3*). (B) Chromosomal views of log2 ratio of % cytosine methylation in mutants compared to a Col control.

Consistent with the lack of a strong TE silencing phenotype for any of the new suppressors of *atxr5/6* (S6 Fig), we observed very limited alterations in DNA methylation in the mutants (Fig 6A). The one exception was the *at-stubl2* mutant (Sample 5, Fig 6A), which showed a relatively large number of DMRs. We attribute this to the ecotype differences between the *at-stubl2-2* Ws background and the control Col ecotype, since *Arabidopsis* ecotypes are known to contain differentially methylated regions [31]. In agreement with this interpretation, *at-stubl2*-2 mutants showed little to no alteration of DNA methylation patterns at the chromosomal level (Fig 6B). *mbd9* mutants were previously reported to show global hypermethylation [24], however, while we observed a relatively higher number of CG-context hypermethylated DMRs for *mbd9-3* mutants (Sample 2, Fig 6A) as compared to the other mutants, analysis of the overall genome levels of DNA methylation suggests this effect on DNA methylation is minor (Fig 6B).

Consistent with lack of strong methylation defects, the mutants isolated from our screen also did not exhibit dramatic morphological defects. This is also consistent with previously published data showing that a triple mutant of all three *SAC3b* related genes in Arabidopsis was reported to have no morphological defects [15] and that the *mbd9* mutant displays only subtle flowering time and branching defects [23].

## Discussion

ATXR5/6 represents a novel link between epigenetic gene regulation and genomic instability because the *atxr5/6* mutant exhibits both de-repression of transposons in pericentromeric heterochromatin, as well as an over-replication defect manifested as the production of excessive DNA from these pericentromeric regions [7]. An open question has been the causal relationship between these two phenomena. *A priori* we can pose three different models of this relationship: 1) *atxr5/6* mutations cause replication defects that indirectly cause transcriptional defects, 2) *atxr5/6* mutations cause transcriptional defects that indirectly cause replication defects, or 3) *atxr5/6* mutations affect replication and transcription independently. Based on the work presented here, we favor model 2 to the exclusion of model 1, but we cannot rule out model 3. Model 1, the model wherein transcriptional defects in *atxr5/6* are due to defects in DNA replication, seems very unlikely based on our previous work characterizing the impact of the loss of DNA methylation pathways on the *atxr5/6* phenotype. This work indicated that the transcriptional silencing phenotype of *atxr5/6* mutants was not dependent on the over-replication phenotype, since DNA methyltransferase mutants suppressed the over-replication phenotype but actually enhanced the transcriptional derepression phenotype of *atxr5/6* [6]. In addition, in the current study we found that irradiation-induced DNA damage caused an upregulation of DNA damage response genes resembling that found in the *atxr5/6 mutant*, but did not cause a transposable element silencing defect. In contrast, the possibility that transcriptional defects in the *atxr5/6* mutant are the cause of the genomic instability defects (model 2) are consistent with the data in the current study. First, by comparing immature flower tissue with cotyledon tissue we observed a co-occurrence of the transcriptional silencing defect and the extra DNA phenotype in cotyledons, both of which were absent in immature flower tissue. Furthermore, the *brca1* mutation acted as an enhancer of both the transposon derepression phenotype and the extra DNA phenotype of *atxr5/6*. Lastly, we performed a screen for mutants that suppress the *atxr5/6* phenotype, and found that every suppressor reduced both the transposon derepression phenotype and the extra DNA phenotype of *atxr5/6*. Thus the transcriptional silencing defects of *atxr5/6* were inseparable from the abnormal pericentromeric DNA content in these mutants, suggesting that the transcriptional misregulation may be the cause of the genome instability phenotype.

If our preferred model to explain the phenotype of *atxr5/6* is correct, there remain many open questions. For instance, it is not clear why ATXR5/6 appears to be exceptional among transcriptional silencers, such as DNA methylation proteins, with regards to a link to aberrant DNA replication. Because ATXR6 is expressed at the G1/S transition of the cell cycle, showing close co-expression with DNA replication licensing factors such as CDT1 and ORC2, we previously speculated that ATXR5/6 and H3K27 monomethylation may act to limit DNA replication initiation, and that extra DNA in the *atxr5/6* mutant was due to inappropriate multiple firing of origins of replication (re-replication), creating “onion skins” [32] of excessive DNA near origins [7]. Although this is still a possibility, because of the tight linkage between transposon upregulation and extra DNA production in *atxr5/6* as well as in our newly identified suppressors, we favor a model in which aberrant G1/S phase transcription in *atxr5/6* leads to replication-transcription conflicts, which ultimately lead the production of excessive DNA in heterochromatin. As part of the normal cell cycle, there must be coordination of DNA replication with transcription, and in both prokaryotic and eukaryotic systems, failure to coordinate DNA transcription and replication results in genome instability [33–35]. This instability can be caused by direct collision between DNA and RNA polymerase complexes as well as by indirect conflicts between the complexes as is the case with R-loop formation by RNA polymerases which can act as a barrier to DNA replication fork progression [21,36]. In both the direct and indirect cases of replication-transcription conflict, the result can be replication fork stalling and collapse [36–38] which in turn leads to the formation of single-stranded DNA and recombinagenic structures that can lead to mutagenic outcomes for the genome [39]. It is thus possible that *atxr5/6* mutations generate genomic instability via the release of transcriptional silencing at a critical point during the cell cycle such as S-phase, which then creates replication-transcription conflicts and hyper-recombinagenic structures that result in the amplification of repetitive DNA in pericentromeric regions. In this way, the timing of transcriptional derepression may differentiate *atxr5/6* mutants from other mutants such as DNA methylation mutants that exhibit loss of TE silencing, but no effect on DNA replication.

A replication-transcription conflict model would be in line with studies of the human [40] and yeast [41,42] genomes where it has been proposed that replication stress can lead to the generation of copy number variants at repetitive DNA. In support of the notion of such a replication-specific silencing function, ATXR5/6 have been characterized as cell-cycle regulated proteins which act with the PCNA proteins normally found at replication forks [43], suggesting that these proteins function during S phase. Furthermore, *ATXR5/6* have been implicated in genetic and epigenetic control of normal rDNA repeat behavior [44], and the ribosomal repeats are known sources of replication-transcription conflict in yeast [45]. The reason for the specificity of the genome instability defect for heterochromatin regions is not known, but it seems possible that the resolution of transcription and replication fork collisions may be more difficult to complete in heterochromatin regions due to the more inaccessible nature of heterochromatin. This could also help explain why DNA methylation mutants suppress the genome instability defect of *atxr5/6* mutants, since severe reduction of DNA methylation would render these regions much less like heterochromatin and more like euchromatin, for instance through reduced levels of the H3.1 histone variant recently shown to be required for over-replication in *atxr5/6* [46].

Our identification of the TREX-2 complex as being necessary for the genomic instability defect in *atxr5/6* mutants also supports the hypothesis of replication-transcription conflict driving the *atxr5/6* genome instability, because components of this complex were isolated in a yeast genetic screen for factors affecting the viability of a strain genetically predisposed to accumulate aberrant replication intermediates [47]. TREX-2 mutants were found to rescue the viability of a replication-deficient strain where replication forks were destabilized in a manner that is phenomenologically similar to our observations of the *trex-2 atxr5/6* triple mutants. In yeast, genetic rescue by the TREX-2 mutants was proposed to act via the loss of topological strain created by the normal gene gating facilitated by TREX-2/THO. Interestingly, this defect depended on transcription but not R-loop formation. In addition, the TREX-2 complex is also required for transcriptional efficiency [17], and TREX-2 was shown to promote RNA Pol II transcription through its interaction with the Mediator complex [16]. This is consistent with our finding that TREX-2 mutants reduce the inappropriate transcription of heterochromatin seen in *atxr5/6* mutants, and suggests that loss of TREX-2 may alleviate replication stress present in an *atxr5/6* genome, which is otherwise undergoing heterochromatic transcription. Similarly, MBD9, which is identified here and which has been characterized as a transcriptional activator of the flowering gene *FLC* [24], likely acts to promote transcription, such that loss of MBD9 would alleviate transcription-induced replication blocks in a manner similar to TREX-2 mutants. Finally, although the function of *At-STUbL2* is not known, since this mutant also reduces the transposon over-expression phenotype of *atxr5/6*, we propose that *STUbL2* acts via similar mechanisms as the TREX-2 and MBD9 mutants, and may encode a transcriptional regulator.

An alternative model to explain the correlation between the transcriptional defects and over-replication defects in *atxr5/6* is that R-loops generated by inappropriately transcribing transposons directly cause mutagenic events leading to excessive DNA production, even in the absence of replication-transcription conflicts [48]. R-loops are formed during the process of transcription where the RNA strand pairs with the complementary DNA strand, leaving the other DNA strand free, and exposing the cell to potentially mutagenic single stranded DNA[21]. If *atxr5/6* mutants fail to properly resolve R-loops in heterochromatin, this could explain the heterochromatin specificity of the excessive DNA phenotype, and also explain why mutants that suppress the transcriptional upregulation of *atxr5/6* also suppress the over-replication defect. Consistent with this model, BRCA1 is known to play a role in the prevention of DNA damage due to transcription associated R-loops [13], and *brca1* mutants enhanced the excessive DNA damage phenotype of *atxr5/6* mutants. Although TREX-2 is also known to be involved in resolving R-loop structures[49], the trex-2 mutants from our screen dramatically reduced the transposon de-repression defect of *atxr5/6* mutants, and therefore would also dramatically reduce the abundance of R-loops. One observation that does not fit well with the R-loop model is that *brca1* mutant enhanced both the magnitude of the transposon over-expression phenotype and the over-replication defect, and it is difficult to understand how failure to resolve R-loop-induced DNA damage would lead to an increase in transcription. Clearly, the mode of action of Arabidopsis BRCA1, TREX-2, and the other suppressors identified here, will be an important question for future studies.

Given the emerging importance of the interaction between epigenome and genome stability for models of disease such as cancer [2,50,51], it will be important to further test the replication-transcription conflict and other models of the *atxr5/6* phenotypes since further understanding of this phenomena may inform other models and systems where loss of transcriptional control leads to genomic instability.

## Materials and Methods

### Genetic strains

All strains used in this study, unless otherwise indicated, were in a Columbia (Col) ecotype background. Details regarding the strain information as well as the generation of the *RAD51pro*::*GFP* line can be found in the Supplemental Experimental Procedures (S1 Text). In addition, S4 Table details the genotypes of lines used in high-throughput sequencing experiments.

### Irradiation

10-day old seedlings were irradiated on plates via exposure to a Cs-137 source following the general experimental design previously described [9].

### EMS mutagenesis

EMS mutagenesis of ~2000 *atxr5/6* seeds carrying the *RAD51pro*::*GFP* transgene was carried out as previously described [52].

### Flow cytometry and FACS

All flow cytometry analysis and FACS was performed as previously described [6]. For cotyledon tissue, cotyledons from at least 20 plants were pooled, whereas for leaf or floral tissue 3 plants were typically pooled.

### Sequencing library generation

The DNA-seq libraries presented in Figs 1 and 2 were generated as previously described [6,7]. The DNA-seq libraries in S3, S5 Figs and Fig 4 were similarly prepared regarding DNA extraction and Covaris shearing, but the libraries were prepared using either the Illumina DNA TruSeq or Nugen Ultralow Ovation kits (see GEO accession GSE77735 for details).

All RNA-seq libraries were prepared using a standard Trizol (Life Technologies) RNA extraction followed by library generation with the Illumina RNA TruSeq kit. All RNA was derived from the cotyledon tissue of >20 plants unless otherwise indicated. For all libraries two biological replicates were performed unless otherwise indicated.

For whole genome bisulfite sequencing libraries, libraries were generated from 3-week-old adult leaf material using the NuGen Ovation Ultralow Methyl-Seq kit before being bisulfite converted with the Qiagen Epitect bisulfite kit using the FFPE protocol. All libraries were sequenced on an Illumina HiSeq instrument.

### Data analysis

Base calls were performed using the standard Illumina pipeline and all reads were aligned to the TAIR10 genome (www.arabidopsis.org). For DNA-seq libraries reads were aligned using the Bowtie aligner [53], for RNA-seq Tophat2 [54]was used, and for whole-genome bisulfite data the BSmap aligner was used [55]. Protein-coding genes were defined as described in the TAIR10 annotation (www.arabidopsis.org) and transposable elements were defined using a previously described list [56] that had been updated to the TAIR10 assembly. All statistical analysis was performed in an R environment. Details of bioinformatics data analysis can be found in the Supplemental Experimental Procedures (S1 Text).

### Data deposition

The sequencing data have been deposited in the Gene Expression Omnibus (GEO) database under accession number GSE77735.

## Supporting Information

S1 Fig RNA-seq of irradiated seedlings yields similar results to previous microarray studies. Venn diagram detailing the significant overlap (P<2.2e-16, Fisher’s Exact Test) of genes identified as up-regulated in the RNA-seq dataset as compared to the previously published microarray study.
(EPS)

S2 Fig Establishment of a GFP-based screen for suppressors of *atxr5/6*. (A) GFP fluorescence of *RAD51pro*::*GFP* in *atxr5/6* cotyledons is lost upon crossing to a Col control. (B) Diagrammatic representation of the mutagenesis, screening, and mapping schema. GFP positive cotyledons are colored green and GFP negative tissues are colored red (due to the autofluoresence of chlorophylls). The *RAD51pro*::*GFP* transgene was maintained at each step by growing plants on selective media (hygromycin). (C) Identification of the *ems_2_37* (*atsac3b-4*) mutant in an M2 family using UV without a band-pass filter and with a band-pass filter (removes chlorophyll autofluoresence). Mutant plants segregated for GFP- cotyledons.
(TIF)

S3 Fig RNA-Seq and DNA-seq confirm the identity of the *ems_2_37* and *ems_2_300* mutations as affecting components of the TREX-2 complex. (A) Pie charts showing the distribution of significantly enriched mutations in the EMS_2_37 (chromosome 3) or EMS_2_300 (chromosome 2) GFP- RNA-seq libraries across the 5 Arabidopsis chromosomes. The “Genome” pie chart gives the distribution of all bp in the Arabidopsis genome. (B) Chromosomal view of the position of significantly enriched mutations in the EMS_2_37/EMS_2_300 GFP- RNA-seq libraries as well as the percent mismatch for each mutation. (C) Screen shot of aligned RNA-seq reads showing intron retention at the *AtSAC3B* gene in the *ems_2_37* (*atsac3b-4*) library. The *atsacb3-4* lesion can be seen as a red mismatch in the sequencing reads at the left intron-exon boundary. (D) Pie charts showing the distribution of significantly enriched mutations for the five Arabidopsis chromosomes from DNA-seq libraries of the *ems_2_37* and *ems_2_300* mutants. (E) Chromosomal distribution and mismatch frequency of significant mutations derived from DNA-seq data of the *ems_2_37* and *ems_2_300* mutants. (F) Gene structure of *AtSAC3B* and *AtTHP1* showing newly identified point mutations from EMS mutagenesis as well as insertional mutants (triangles) used for complementation and downstream analysis. Exons are represented by black boxes.
(EPS)

S4 Fig Flow cytometry confirm the identity of the *ems_2_37* and *ems_2_300* mutations as affecting components of the TREX-2 complex. (A) Flow cytometry of nuclei counts as in Fig 1A showing that the *atsac3b-3* insertional allele suppresses the *atxr5/6* extra-DNA phenotype similar to the mapped point mutations. (B) The insertional *atthp1-1* allele suppresses the *atxr5/6* extra-DNA phenotype. (C) Complementation analysis showing the TREX-2 insertional alleles fail to complement the EMS alleles because the *atxr5/6* extra-DNA phenotype is suppressed. Also shown is the non-complementation of the *ems_2_209* line crossed to *ems_2_37*, confirming the identity of *ems_2_209* as *atsac3b-5*. Also shown is a control cross of *ems_2_37* crossed to *atxr5/6* grown in parallel to show the re-emergence of the *atxr5/6* extra-DNA phenotype in the F1.
(EPS)

S5 Fig Mapping and complementation analysis of the *ems_2_325* mutation that identifies At-STUbL2 protein. (A) Pie charts and chromosomal views as shown in S3A and S3B Fig showing the distribution of significantly enriched mutations in *ems_2_325* (GFP-) plants identified in RNA-seq and (B) DNA-seq data. (C) Gene (top) and protein (bottom) structure of At-STUbL2 showing the newly identified point mutation from EMS mutagenesis as well the insertional mutant (triangles) used for complementation and downstream analysis. For the gene structure the black boxes represent exons, and for the protein structure gray boxes represent pfam domains. (D) No insertional allele of *At1g67180* exists in the Col ecotype used for all other lines in this study for complementation purposes, so we obtained an insertional mutant (FLAG_430E03) isolated in the Ws ecotype [57]. The hybrid nature of the genome resulting from complementation crosses between the Ws allele and our *ems_2_325* line made direct comparison to the control *atxr5/6* line difficult and we also noted that the *atxr5/6* extra-DNA defect was severely reduced in the 50% Ws *atxr5/6* plants regardless of the *At1g67180* genotype. The graph shows flow cytometry showing that the *atstubl2-2* insertional allele suppresses the *atxr5/6* extra-DNA phenotype as well as partial suppression of the extra-DNA phenotype in the control 50% Ws line. (E) Complementation analysis comparing crosses between *ems_2_325* line crossed to the *atstubl2-2 atxr5/6* triple mutant (showing strong non-complementation) with a control cross to *atxr5/6*. (F) Quantitation of the 16C peak, shown in S5E. Coefficient of variation (CV) of the F1 complementation material shows a slight reduction in extra-DNA (small CV value) for the *atstubl2-2 atxr5/6 x ems_2_325* plants as compared to control *atstubl2-2 atxr5/6 x atxr5/6* plants. (G) To overcome the confounding factor of the genetic background, we performed RNA-seq on F1 plants resulting from a cross of a *FLAG_430E03 atxr5/6* triple mutant (50% Col; 50% Ws ecotype) with pollen from either an *atxr5/6* mutant or the *ems_2_325* line. The resultant progeny were all 75% Col, 25% Ws in genome composition and the RNA-seq results showed clear suppression of *atxr5/6* transposon expression and irradiation-induced genes in those plants carrying both the insertional FLAG_430E03 allele and the *ems_2_325* allele relative to plants heterozygous for a functional copy of At1g67180. The box plots show RNA-seq RPKM values for *atxr5/6*-induced TEs and (H) irradiation-induced genes for *ems_2_325* complementation material showing non-complementation (*atxr5/6* suppression) by *atstubl2* alleles. (I) Chromosomal views of the log2 ratio of normalized RNA-seq reads between the non-complementing *atstubl2-2 atxr5/6* x *ems_2_325* F1 material compared to a control *atstubl2-2 atxr5/6* x *atxr5/6* control F1 cross.
(EPS)

S6 Fig Suppression of *atxr5/6* transcriptional phenotypes by newly identified suppressors of *atxr5/6*-induced extra-DNA. Aggregation of data shown throughout this study for comparative purposes showing cotyledon RNA-seq RPKM values for *atxr5/6*-induced TEs and irradiation-induced genes for the newly identified *atxr5/6* suppressors.
(EPS)

S1 Table Genes and transposons upregulated compared to wild type in atxr5/6 cotyledons and flowers. (XLSX)

S2 Table Genes and transposons upregulated upon irradiation (100Gy treatment) in wild type seedlings as compared to control seedlings. (XLSX)

S3 Table Gene Ontology Analysis of Top 200 Genes Co-expressed with At-STUbL2. (XLSX)

S4 Table Sample manifest detailing the genotype, replicate number and name as present in GEO database submission for high-throughput sequencing experiments detailed in the paper. (XLSX)

S1 Text Supplemental Experimental Procedures. (DOCX)
