# Supplementary figures and images for: Identification of Multiple Proteins Coupling Transcriptional Gene Silencing to Genome Stability in Arabidopsis thaliana
Source: PLoS Genet. 2016 Jun 2;12(6):e1006092. doi: 10.1371/journal.pgen.1006092 (PMC4890748; doi:10.1371/journal.pgen.1006092)

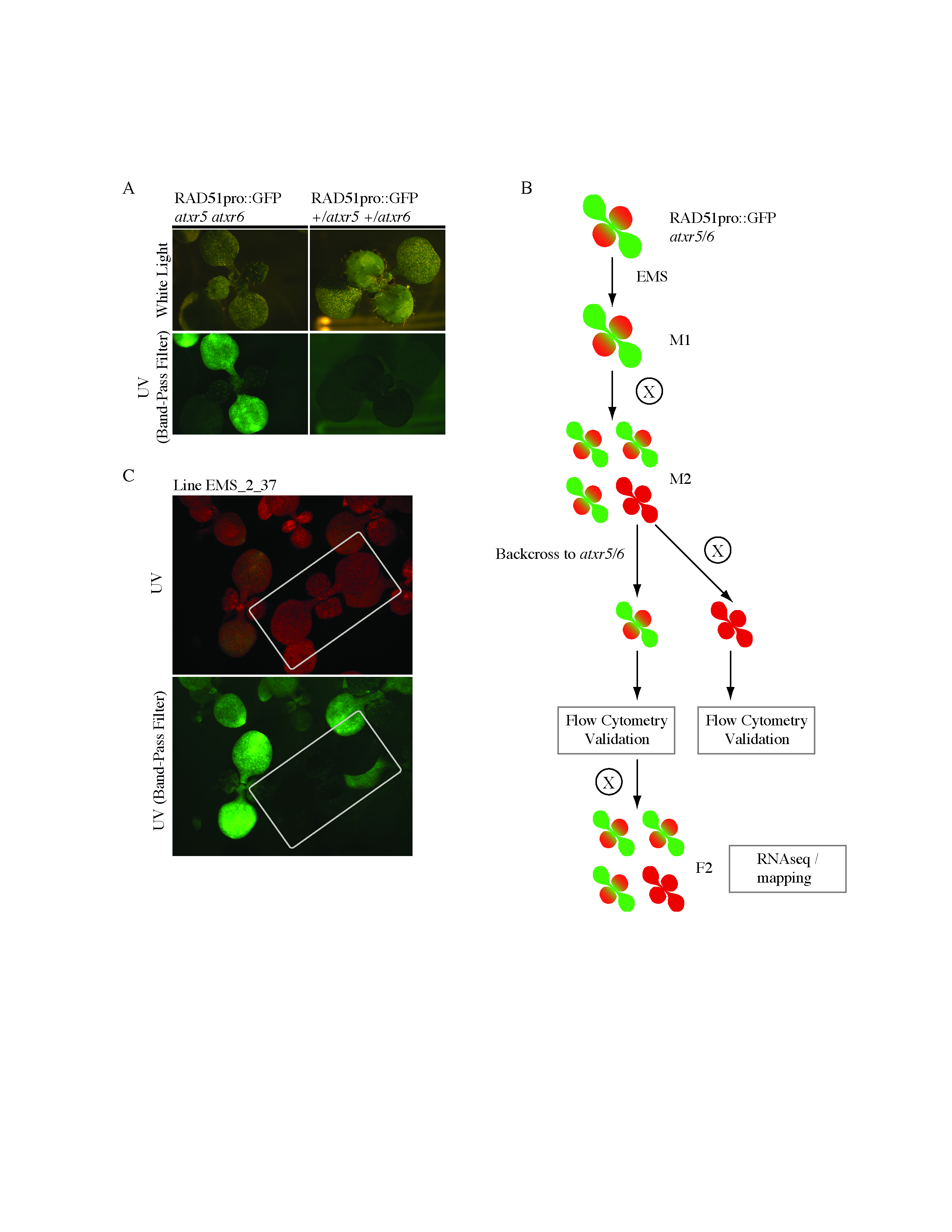

Supplement: S2 Fig — (A) GFP fluorescence of RAD51pro::GFP in atxr5/6 cotyledons is lost upon crossing to a Col control. (B) Diagrammatic representation of the mutagenesis, screening, and mapping schema. GFP positive cotyledons are colored green and GFP negative tissues are colored red (due to the autofluoresence of chlorophylls). The RAD51pro::GFP transgene was maintained at each step by growing plants on selective media (hygromycin). (C) Identification of the ems_2_37 (atsac3b-4) mutant in an M2 family using UV without a band-pass filter and with a band-pass filter (removes chlorophyll autofluoresence). Mutant plants segregated for GFP- cotyledons. (TIF) [file pgen.1006092.s002.tif]
