# Supplementary material for: Identification of Multiple Proteins Coupling Transcriptional Gene Silencing to Genome Stability in Arabidopsis thaliana
Source: PLoS Genet. 2016 Jun 2;12(6):e1006092. doi: 10.1371/journal.pgen.1006092 (PMC4890748; doi:10.1371/journal.pgen.1006092)
Supplement: S1 Text — (DOCX) [file pgen.1006092.s011.docx]

**SUPPLEMENTAL EXPERIMENTAL PROCEDURES**

**Generation of the *RAD51pro::GFP* transgene**

The *RAD51pro::GFP* strain was created using the standard agrobacterium-mediated floral dip transformation on *atxr5/6* mutants [1]. The GFP protein [1] was fused to sequence corresponding to the upstream region (1 kb of sequence 5’ of the start codon) of the *RAD51* gene. The resulting transgene also carried a hygromycin resistance marker that allowed for plant selection. Primer sequence used to amplify this sequence is found in below.

**DNA-seq analysis**

The flower, cotyledon, and 16C sorted nuclei libraries presented in Fig 1 and 2 were all sequenced at 100 bp length. Due to poor quality of the 3’ end of these reads, the flower and cotyledon reads were trimmed to 50 bp in length while the 16C libraries were trimmed to 80 bp in length for downstream analysis. Flower/cotyledon reads were mapped to the TAIR10 genome using Bowtie [2] allowing for 1 mismatch, while 16C reads were allowed 2 mismatches given their greater length. For all mappings, only uniquely mapping reads were retained. RPKM of genes/TEs was calculated as (((# of reads overlapping a feature)/(total aligning reads for that library))/length of the feature)*1000000*1000. For generation of chromosomal views, 100 kb regions of low coverage (coverage below the 5^th^ percentile) in either library of the comparison were excluded from analysis. As the sum of log2 ratios between any library should total to 0, we chose to center the log2 ratio on the average ratio of any two compared libraries on the first 5 Mbp of chromosome 1. This avoids the computational artifact of giving a negative ratio of coverage for *atxr5/6* mutants compared to Col over euchromatic regions, something we do not believe is biologically relevant.

**RNA-seq analysis**

All RNA-seq reads were mapped to the TAIR10 genome build using Tophat2 [3], allowing for 1 mismatch and retaining only uniquely mapping reads. RPKM values were calculated as (((# of reads overlapping a feature)/(total aligning reads for that library which overlap with mRNA encoding regions))/length of the feature)*1000000*1000. To call genes or TEs as up-regulated in a given RNA-seq library, that library must have had two biological replicates and the gene or TE must have shown 4-fold up-regulation as compared to Col in both biological replicates at a False Discovery Rate (FDR) less than 0.01. FDRs were estimated using a Benjamani-Hochberg adjustment of P-values calculated using a Fisher Exact Test (FET).

**EMS-induced mutation mapping via RNA-seq or DNA-seq analysis**

Mapping of EMS-induced genetic lesions was carried out under the operating assumption that EMS treatment would induce a large number of unique mutations in each line sequenced. Following backcross of the EMS mutant to an *atxr5/6* plant and resegregation of the EMS mutants in the F2 generation, any given EMS mutation would be expected to be present at the level of 25% in a population of the F2 mutants. This hypothesis would fail to hold true for mutations linked to the causal EMS mutation, which would therefore be over-represented in the sequencing data at levels exceeding 25% of the reads for a given position.

To identify these over-represented mutations, we first identified candidate mutations in the RNA-seq or DNA-seq data by considering any bp position that had at least 2 mismatches. The candidate mutations were then compared to candidate mutations called for all the RNA-seq or DNA-seq libraries generated from EMS lines as well as control RAD51pro::GFP and Col lines. A candidate mutation was considered unique if it had >=4 mismatches for only one library. These unique candidate mutations were considered significantly enriched in a library, if the mismatched base pair was present at a level greater than 25% of all base pairs sequenced at that position (P<0.01 , Binomial Test). The resulting significantly enriched unique mutations were then visually assessed via chromosomal view plots for clusters that might be indicative of regions of linkage. Following identification of these regions, mutations were scanned for mutations that fell within gene coding regions that were predicted to cause splice site/nonsense/ or missense mutations. These candidate mutations were then chosen for downstream RNA-seq and complementation analysis.

**Whole-genome bisulfite sequencing analysis**

DMRs were identified for the newly identified suppressors of *atxr5/6 f*ollowing alignment of the whole-genome bisulfite sequencing reads to the TAIR10 genome [4]. To identify DMRs, we used methods as previously described [5] with the following modifications: 1) Minimum coverage for a cytosine to be considered in DMR calling was 5X, 2) At least 5 cytosines of a given context must be present in a DMR for that context, 3) Minimum absolute difference in percent methylation to be considered a DMR for CG, CHG, and CHH contexts was 50%, 25%, and 15%, respectively. A Benjamani-Hochberg estimated FDR of <0.01 based on a FET P-value was required to be called as a DMR. Comparisons between the mutant libraries and three Col (wild type) libraries were carried out. To be retained for downstream analysis, a DMR must have been called for a given mutant library in comparison to all three of the Col libraries. The Col samples used included one library generated in parallel and two previously published Col methylomes (GEO samples: GSM1193638 and GSM881756). For the analysis of *met1* in Figure 6, a previously published whole-genome bisulfite sequencing library was used (GSM981031), although the reads from this library were downsampled (to ~50 million reads) to match the coverage of libraries generated in this study, and were realigned and processed using the same parameters as described above.**Primers Used to Amplify the *RAD51* promoter sequence for the *RAD51pro::GFP* transgene.**

| Primer Name | | Sequence | Description |
| --- | --- | --- | --- |
| JP7488 | CTGGGTTTCTTCATCGTCTTG | | Reverse primer directly upstream of RAD51 start codon |
| JP7515 | CACCGTTGACATTCAAACACCTAGGTATC | | Forward primer ~1kb upstream of RAD51, with CACC tag added for Gateway Cloning into P-ENTR D TOPO (Life Technologies) |

**Genetic stocks used in this study**

| Allele | EMS/T-DNA identifier | Gene Target | Ecotype |
| --- | --- | --- | --- |
| *atxr5* | SALK_130607 | *AT5G09790* (ATXR5) | Col |
| *atxr6* | SAIL_240_H01 | *AT5G24330* (ATXR6) | Col |
| *atbrca1-1* | SALK_014731 | *AT4G21070* (AtBRCA1) | Col |
| *atsac3b-3* | SALK_065672 | *AT3G06290* (AtSAC3B) | Col |
| *atsac3b-4* | ems_2_37 | *AT3G06290* (AtSAC3B) | Col |
| *atsac3b-5* | ems_2_209 | *AT3G06290* (AtSAC3B) | Col |
| *atthp1-1* | SAIL_82_A02 | *AT2G19560* (AtTHP1) | Col |
| *atthp1-5* | ems_2_300 | *AT2G19560* (AtTHP1) | Col |
| *bru1-4* | SALK_034207 | *AT3G18730* (BRU1) | Col |
| *fas2* | SALK_033228 | *AT5G64630* (FAS2) | Col |
| *mbd9-3* | SALK_039302 | *AT3G01460* (MBD9) | Col |
| *mbd9-4* | ems_2_129 | *AT3G01460* (MBD9) | Col |
| *atstubl2-1* | ems_2_325 | *AT1G67180* (STUbL2) | Col |
| *atstubl2-2* | 430E03 (FCA227) | *AT1G67180* (STUbL2) | Ws |
| *met1-3* | CS16394 | *AT5G49160* (MET1) | Col |

**REFERENCES**

1. Moissiard G, Cokus SJ, Cary J, Feng S, Billi AC, Stroud H, et al. MORC family ATPases required for heterochromatin condensation and gene silencing. Science. 2012;336: 1448–51. doi:10.1126/science.1221472

2. Langmead B, Trapnell C, Pop M, Salzberg SL. Ultrafast and memory-efficient alignment of short DNA sequences to the human genome. Genome Biol. Nature Publishing Group, a division of Macmillan Publishers Limited. All Rights Reserved.; 2009;10: R25. doi:10.1186/gb-2009-10-3-r25

3. Kim D, Pertea G, Trapnell C, Pimentel H, Kelley R, Salzberg SL. TopHat2: accurate alignment of transcriptomes in the presence of insertions, deletions and gene fusions. Genome Biol. 2013;14: R36. doi:10.1186/gb-2013-14-4-r36

4. Xi Y, Li W. BSMAP: whole genome bisulfite sequence MAPping program. BMC Bioinformatics. 2009;10: 232. doi:10.1186/1471-2105-10-232

5. Stroud H, Greenberg MVC, Feng S, Bernatavichute Y V, Jacobsen SE. Comprehensive analysis of silencing mutants reveals complex regulation of the Arabidopsis methylome. Cell. 2013;152: 352–64. doi:10.1016/j.cell.2012.10.054
